# De-identification of electronic health record using neural network

**DOI:** 10.1038/s41598-020-75544-1

**Published:** 2020-10-29

**Authors:** Tanbir Ahmed, Md Momin Al Aziz, Noman Mohammed

**Affiliations:** Department of Computer Science, University of Manitoba, Winnipeg, Canada

**Keywords:** Biomedical engineering, Health policy

## Abstract

According to a recent study, around 99% of hospitals across the US now use electronic health record systems (EHRs). One of the most common types of EHR is the unstructured textual data, and unlocking hidden details from this data is critical for improving current medical practices and research endeavors. However, these textual data contain sensitive information, which could compromise our privacy. Therefore, medical textual data cannot be released publicly without undergoing any privacy-protective measures. De-identification is a process of detecting and removing all sensitive information present in EHRs, and it is a necessary step towards privacy-preserving EHR data sharing. Over the last decade, there have been several proposals to de-identify textual data using manual, rule-based, and machine learning methods. In this article, we propose new methods to de-identify textual data based on the self-attention mechanism and stacked Recurrent Neural Network. To the best of our knowledge, we are the first to employ these techniques. Experimental results on three different datasets show that our model performs better than all state-of-the-art mechanism irrespective of the dataset. Additionally, our proposed method is significantly faster than the existing techniques. Finally, we introduced three utility metrics to judge the quality of the de-identified data.

## Introduction

The advent in computational power and low storage cost has created the avenue for a systematic electronic collection of health information about patients. These records mostly contain patient information such as medical history, medication orders, vital signs, laboratory results, radiology reports, physician and nursing notes, etc. Recent studies suggest that these integrated electronic health records can improve patient safety against medication error and adverse drug events, help with the short-term preventive care and near-term chronic disease management, and save the overall healthcare cost^1^. In hope of utilizing these benefits, The Health Information Technology for Economic and Clinical Health Act was signed as a law in 2009, designed to ensure access to health care facilitated by Electronic Health Record (EHR) systems. In 2016 about 95% of all eligible hospitals and 62% of all office-based physicians were reported to use Electronic Health Record systems^2^. This widespread adoption of the Electronic Health Record system created valuable medical data worth studying.

However, medical researcher and organizations are not free to use EHRs as they contain sensitive patient identifying information, which is commonly private. To preserve the patients’ confidentiality, the U.S. Health Insurance Portability and Accountability Act (HIPAA) requires that 18 re-identifying categories of information to be removed from medical records before they are disseminated^3^. These confidential information categories are referred to as the Protected Health Information (PHI) which include patients name, their profession, unique identifying numbers like social security or medical number etc.

**Privacy requirement.** In this paper, we study the problem of De-identification, which is identifying (and removing) the PHI instances outlined by HIPAA^4^ from unstructured textual medical records. These medical records are reported by a physician, nurse or lab technician, and contain sensitive information of patients such as patients’ family and social history, medical encounters, demographics, etc. Notably, as this is a free-form text without any predefined structure, it solely depends on the practitioner (i.e., doctor, nurse) how s/he writes the patient or nursing notes. Therefore, we follow the ‘HIPAA Safe Harbour Method’^5^ to detect and sanitize predefined categories (i.e., names, dates, contact numbers etc.) of sensitive information.

**Related works.** De-identification is the necessary step for making the EHRs available for research or public usage, and it has attracted substantial interest in the research community. Over the last decades, a considerable amount of effort has been devoted to this problem, including the use of human annotators to manually de-identify the EHRs. Douglass et al.^6^ reported a cost of $50/hour where human annotators read around 20000 words/hour. At this rate, a dataset having 100 million words would cost an astonishing amount of $250,000. Nematullah^7^ reported the recall value ranged from 63 to 94% depending on the annotators when he asked 14 annotators to de-identify approximately 130 patient notes. Therefore, manual de-identification, which is also prone to errors, is deemed a costly and time-consuming approach for de-identification.

The shortcomings of manual de-identification formed a natural progression towards automated systems, and there are several proposed approaches to de-identify EHRs automatically. These existing automated de-identification systems are predominantly rule-based^6^ or employ machine learning algorithms^8^. The rule-based systems rely primarily on the word patterns captured using pre-defined regular expressions and dictionary lookups and do not require any labeled data. Although these systems are easy to develop, they do not generalize well as the rules have to be fine-tuned for each dataset. Also, rule-based systems do not take the context of the words into account. The deficiencies of the rule-based systems are mostly mitigated by the use of supervised machine learning approaches. These machine learning systems are trained as a classifier model, where each word is labeled as PHI or not, often identifying the different PHI types as well. With all their efficacies and conveniences, these systems still have one common problem: the dependency on handcrafted features, which are challenging and time-consuming.

Recent approaches in Natural Language Processing (NLP) tasks such as Named-entity Recognition (NER) and Parts of Speech (POS) tagging^9^ using non-linear neural networks have shown promising results without any handcrafted features or rules. The features in these systems are learned automatically with other parameters of the network during training on a labeled dataset. However, all of these approaches used the variants of Recurrent Neural Networks (RNNs). More specifically, using bidirectional Long Short Term Memory (LSTMs) for determining the correlation between the words in unstructured textual EHRs. Since 2017, several new models for neural language modeling have been proposed^10,11^, which are built entirely with attention layers, without convolution or recurrence. These papers report new state-of-the-art F1 scores for various language modeling task, including NER. Theoretically, the new self-attention based approaches should also improve the de-identification results. In this paper, we evaluate the performance of self-attention model on the de-identification task. To the best of our knowledge, we are the first to employ self-attention mechanism to the de-identification problem. We also propose an architecture employing multiple neural networks for de-identification and analyze them in different settings. (More on related works in the supplementary information).

**Contributions. ** We proposed an architecture to de-identify EHRs using different deep neural networks: i) bi-directional Gated Recurrent Units (GRU)^12^ ii) Stacked Recurrent Neural Network (RNN)^13^ structure combining GRUs and/or Long Short Term Memory (LSTM)^14^ units and iii) finally, replacing the recurrent units with only a self-attention based mechanism. Notably, these approaches employ two different embedding schemes (fixed and dynamic) that are not considered by earlier attempts. We also conducted multiple experiments to compare the results with the existing state-of-the-art approach^15^ on three standard de-identification datasets. The main contributions of this article are summarized as follow:

- **Utility Metric.** We introduced two general purpose utility metrics and one specific application to judge the quality of the de-identified data. To the best of our knowledge, this is the first article to introduce utility metrics for a de-identified textual dataset. Experimental results showed that our de-identified documents have comparable utility in different settings compared to the state-of-the-art.
- **Scalability.** We analyzed the performance of self-attention mechanism and compared the results to other RNN based models. Experimental results show that the proposed model requires significantly less time to train and predict compared to the existing models. This is significant since deep learning-based algorithms are computationally expensive and often do not scale well for larger datasets. Experiments on three different datasets show that our model can perform well in both smaller (i2b2 2014) and larger (Nursing Note, MIMIC-III) datasets.
- **Accuracy.** Our proposed methods achieve 85.9% F1-score ($4\%$ improvement) compared to the state-of-the-art method^15^ for the Nursing Notes. Furthermore, our methods outperform the predecessors by attaining 99.97% F1-score ($0.03\%$ improvement) and 98.22% F1-score (0.4% improvement) on the MIMIC-III and i2b2 dataset, respectively.

## Results

We evaluated our architecture on three different datasets: i2b2^16^, Nursing Note^17^ (aka MIMIC-II) and MIMIC-III^18^. The i2b2 is the benchmarking dataset due to its primary usage over a de-identification competition held in 2014. The dataset has categorized the EHRs into train, validation, and test sets where the annotations were done manually. On the other hand, the Nursing Note or the MIMIC-III both went through some form of computer-assisted de-identification. The i2b2 dataset was much smaller compared to the other two. MIMIC-III is much larger (60k patient admission data) corpus compared to i2b2 (1304 documents). We considered 4,441 discharge summaries from MIMIC-III averaging around 1,387,990 (1.3 M) words. $20\%$ (891 EHRs) of the dataset was selected for testing, whereas the training set was split 80 : 20 for training and validation, respectively. We used the BIOES tagging scheme instead of standard BIO2, as previous studies have reported meaningful improvements with this scheme. We present the corpora statistics regarding the number of PHI instances in the supplementary documents. Furthermore, the Nursing Note dataset has 2,434 documents with 1,826 PHI instances. We split the dataset as the MIMIC-III dataset in 80:20 ratio to create the training (1948 documents) and test (486 documents) sets. The experimental results are presented into three categories: accuracy in predicting PHI tokens, utility analysis of the de-identified data, and the execution time of the proposed models.

### Accuracy

To assess the accuracy/performance of the model, we computed the precision (P), recall (R), and F1-score of our architecture which are defined as $P = TP/(TP+FP)$, $R = TP/(TP+FN)$ & $F1 = 2\times precision\times recall/(precision+recall)$, respectively. Here, *TP* is the number of PHI instances our model correctly labeled a token as a PHI, *FP* is the number of non-sensitive tokens our model labeled as a PHI & *FN* is the number of sensitive tokens (PHI) our model labeled a token as not a PHI. Intuitively, precision is the proportion of predicted named entities that are ground truth labels, recall is the proportion of ground truth named entities that are correctly predicted, and F1-score is the harmonic mean of precision and recall.

Table 1 shows the best results we have found for each of our models on the three datasets. The results reported in Table 1 are evaluated based on the detection of PHI tokens vs non-PHI tokens (i.e., binary HIPAA token-based evaluation). We used Dernoncourt et al.’s^15^ model as our performance benchmark as this work has a better performance than any existing models for the i2b2 dataset. For the i2b2 dataset, we reported the results directly from their paper. However, their preprocessed MIMIC-III dataset is not publicly available, and it was not possible to reproduce the dataset solely from their paper. Hence, for a fair comparison, we had to reproduce the results using the publicly available implementation of their architecture. We used the pre-trained models with binary evaluation method outlined in their implementation and reported the best result we found. Dernoncourt et al.^15^ did not report their results on Nursing Note. We used their best performing pretrained model and ran the model until convergence as we did for the MIMIC-III dataset, and reported the results. From Table 1, we can see that Khin et al.^19^ has better performance ($81.2\%$ F1 Score) than Dernoncourt^15^ model ($77.0\%$ F1 Score). Hence, we regard Khin et al.^19^ as our benchmark for the Nursing Note dataset. However, Khin et al.did not report their results on MIMIC-III dataset and their implementation was not publicly available at the time this paper is written. Thus, the columns for the MIMIC-III dataset of this model are left blank in Table 1.

**Table 1:** Precision (P%), Recall (R%) and F1 (%) comparison for each dataset.

Model	i2b2 2014^16^			MIMIC-III^18^			Nursing notes^17^		
	P(%)	R(%)	F1(%)	P(%)	R(%)	F1(%)	P(%)	R(%)	F1(%)
Dernoncourt et al.^15^	97.920	97.835	97.877	99.91	99.97	99.94	86.10	69.70	77.00
Khin et al.^19^	98.300	97.370	97.830	–	–	–	**91**.**40**	74.30	81.20
GRU (Proposed)	98.746	95.859	97.281	99.946	**100**.**00**	**99**.**973**	81.82	66.23	73.21
GRU-GRU (Proposed)	**99**.**011**	95.124	97.028	99.939	99.994	99.967	80.00	71.00	75.23
LSTM-GRU (Proposed)	98.749	95.278	96.982	99.940	99.998	99.969	85.14	68.18	75.72
Self-attention (Proposed)	98.031	**98**.**410**	**98**.**220**	**99**.**957**	98.788	99.369	89.20	**82**.**90**	**85**.**90**

Bold values indicate heighest values of their respective columns.

The results in Table 1 show that our attention model has better F1 score (98.220%) than all our RNN based models on the i2b2 dataset, and it is 0.343% more than the reported value by the state-of-the-art^15^. The attention model also has a 0.111% higher precision and 0.575% recall value than^15^. Surprisingly, the GRU model has the best F1 score (97.281%) than the other RNN models, although it falls behind of the attention model by 0.939%. Among the stacked RNN models, the GRU-GRU model has a 0.046% F1 score gain over the LSTM-GRU model. The GRU-GRU model has a higher precision value than the self-attention model, however, a very low recall value (95.124%) leads to a lower (97.028%) F1 score. Furthermore, the GRU, GRU-GRU, and LSTM-GRU model have 98.746%, 99.011%, and 98.749% precision score, respectively. All these precision values are higher than the benchmark approach.

Results on the MIMIC-III dataset are slightly different. The prior model^15^ performed better on this dataset compared to the i2b2 dataset. Their F1 score is 99.94% that leaves a limited margin for improvement. However, our proposed models still managed to incur improvements over the state-of-the-art model^15^. Unlike the i2b2 dataset, the GRU model has the best F1 score (99.973%). Despite having the best precision score, (99.957%) a 1.212% lower recall value resulted in 99.369% F1 score for the attention model. In addition to the GRU model, the GRU-GRU and the LSTM-GRU model both have better F1 score (99.967% and 99.969% respectively) than^15^. Please be noted that Khin^19^ did not have their implementation publicly available. Therefore, we are unable to report the results for their model on the MIMIC-III dataset. In summary, our GRU, GRU-GRU, and LSTM-GRU model have 0.033%, 0.027% and 0.029% higher score than the baseline model^15^.

Now, our self-attention model outperforms both models by at least 4.5% in the Nursing Note dataset. However, our proposed RNN and stacked RNN models performed underwhelmingly in this dataset compared to other datasets. Even though all of the three models (GRU, GRU-GRU & LSTM-GRU) have precision values over $80\%$, the RNN based models have lower recall values compared to Khin et al.^19^ and our self-attention model.

The i2b2 2014 dataset is the standard dataset for evaluating any de-identification system. For further analysis of our model we thus experimented on this dataset. The results presented in Table 2a,b show results found only in the i2b2 dataset. In Table 2a, we study the effect of character embedding and different dropout values on our GRU and self-attention model. First, we evaluated our GRU model without any character embedding (CE) at a $50\%$ dropout value. Then we added the character embedding at the same dropout rate. Then we incrementally decreased the dropout value to $25\%$ and $0\%$. F1-scores for these two dropout rates were $95.791\%$ and $96.817\%$, respectively, which is lower than the F1-score at $50\%$ dropout rate ($97.281\%$).

The self-attention model uses dynamic embedding. Therefore, there was no requirement to use character embedding with self-attention. Now, the highest F1-score was found for a $10\%$ dropout rate. Although, at higher dropout rates ($25\%$ & $50\%$), the recall value increases, the precision values continue to drop. We report the results at $25\%$ dropout in Table 1 as at this rate, the model has fairly higher values for both recall ($98.41\%$) and precision ($98.03\%$). Table 2a also shows the impact of different mechanisms in the label decoding layer, for the GRU and the self-attention model. For both of these models, when a CRF algorithm is used to optimize the detection of PHI instances, the F1-scores are higher ($0.81\%$ and $0.098\%$, respectively) compared to when a softmax function (detailed in “Label decoding layer” section) was used.

To improve the recall value of the self-attention model, during training we modified the loss function. We introduced a new hyperparameter, $\delta _{p}$ to control the penalty for low recall value. (“Loss function” section gives details about the formulation of this hyperparameter.) Table 2b shows the results we found for different values of $\delta _{p}$ for the i2b2 dataset. For $\delta _{p}=10$, the recall value improves 0.375% over the values for $\delta _{p}=1$. This improvement indicates that by penalizing the network with respect to the false negative prediction rate, we can improve the recall value. Although the precision value drops by 0.247%, the recall bears more significance than the precision for the de-identification.

**Table 2:** (a) Effect of Dropout Rate, character embedding (CE), CRF and Softmax layer on GRU and Self-attention(SAT) model i2b2 dataset, where P (%), R (%), F1 (%) denote precision, recall, F1 score, respectively. (b) Effect of Penalty $\delta _{p}$ on the SAT for i2b2 2014 dataset.

Model	CE	CRF	Softmax	Dropout	P(%)	R(%)	F1(%)
**(a)**							
GRU	$\times$	$\checkmark$	$\times$	0.50	97.859	84.560	90.725
GRU	$\checkmark$	$\checkmark$	$\times$	0.00	98.544	93.187	95.791
GRU	$\checkmark$	$\checkmark$	$\times$	0.25	98.677	95.026	96.817
GRU	$\checkmark$	$\checkmark$	$\times$	0.50	**98**.**746**	**95**.**859**	**97**.**281**
GRU	$\checkmark$	$\times$	$\checkmark$	0.50	97.768	95.206	96.470
SAT	-	$\checkmark$	$\times$	0.10	**98**.**705**	97.923	**98**.**313**
SAT	–	$\checkmark$	$\times$	0.25	98.031	98.410	98.220
SAT	–	$\times$	$\checkmark$	0.25	98.854	97.400	98.122
SAT	–	$\checkmark$	$\times$	0.50	93.782	**98**.**559**	96.111

${\delta _{p}}$	**P(%)**	**R(%)**	**F1(%)**				
**(b)**							
1	99.046	97.131	98.079				
3	98.283	97.437	97.858				
5	**99**.**060**	97.269	**98**.**156**				
10	98.799	97.506	98.148				
15	99.056	95.720	97.360				

Bold values indicate heighest values of their respective columns.

### Utility analysis

To measure the utility of the de-identified EHRs, we propose two general-purpose utility metric: the Bilingual Evaluation Understudy (BLEU)^20^ and Topic Modeling and one classification application using the de-identified data as a specific purpose utility metric. We discussed the evaluation method of the classification application in “Classification application” section. Now, to analyze the results for BLEU score Topic modeling evaluation, we calculated two reference scores:

- *Baseline*: To the best of our knowledge, Dernoncourt et al.^15^ reports the state-of-the-art results for de-identification. We calculated the BLEU scores and estimated the topics from the de-identified documents by^15^, which serves as the baseline for our utility evaluation.
- *Ground-truth (GT)*: For our second reference score, the BLEU scores and Topic Modeling estimations were done for the ground truth de-identified documents. These documents contained the original labels of the PHI instances. Hence, if we calculate the precision or recall for these documents, we will always get $100\%$.

Before discussing our results on different utility metrics, we need to examine the relationship of utility with respect to precision and recall (especially, FP and FN). Notably, in a de-identification problem, privacy relies only on recall (or TP and FN) as higher recall denotes that the model was more successful in identifying PHI instances offering better privacy. However, the utility is not as straightforward as it takes FP into account as well. When a model wrongfully labels a token as PHI (=FP), it is sanitized and removed in the de-identified document. Similarly, if the same model classifies a PHI token as non-sensitive (=FN), it gets to stay in the de-identified document (privacy breach). Now, higher values of FPs (lower precision) will dictate more tokens to be missing from the document whereas, higher FNs (lower recall) will increase the token count. Since the utility of the de-identified document relies on its tokens, we need to consider both precision and recall while analyzing it.

**Table 3:** BLEU score comparison in the i2b2 dataset for different *n*-grams. Column BLEU-1, BLEU-2, BLEU-3 list the scores for $n=1,2$ and 3 where P (%), R (%), F1 (%) denote precision, recall, F1 score, respectively.

Method	P(%)	R(%)	F1(%)	BLEU-1	BLEU-2	BLEU-3
Ours	98.03	98.41	98.22	0.923	0.912	0.786
Baseline	97.92	97.84	97.88	0.900	0.887	0.759
GT	100.0	100.0	100.0	0.898	0.885	0.757

**Table 4:** Percentage of phrase and word matches for 5 topics each with 30 words for i2b2 dataset.

Method	P(%)	R(%)	F1(%)	Topic1	Topic2	Topic3	Topic4	Topic5	Overall
Ours	98.031	98.410	98.22	86.6	43.3	60.0	36.7	60.0	84
Baseline	97.920	97.835	97.88	70.0	60	46.7	16.7	46.7	84
GT	100.00	100.00	100.0	86.7	60.0	63.3	60	70.0	83.3

#### BLEU scores

BLEU used different *n*-grams to output scores, which essentially reveals how similar two sentences are in a document. In Table 3, we show the BLEU scores where the ground-truth (GT) documents contained all tokens except the PHI identifiers; hence, the $100\%$ precision and recall value. As the maximum number of removable tokens are sanitized from the GT documents, its utility should be the lowest while compared with the original document (with all tokens). Therefore, the BLEU scores presented for GT is the maximum achievable utility for a fully private de-identified data.

#### Topic modeling

We used Latent Dirichlet Allocation (LDA)^21^ as the probabilistic model for our second utility metric. LDA is an unsupervised ML algorithm that clusters relevant words to a topic. The number of topics was defined according to the coherence score as we experimentally set it as 5. Hence, our LDA model generated 5 different topics, each with 30 words (total 150 words) from the original (with PHI), GT, Baseline, and our de-identified corpus. Table 4 shows the ratio of word frequency in the de-identified documents compared to the original document for the individual 5 topics. We utilized the 30 words from each topic to get the percentage of matches between the original and our de-identified dataset. This matching percentage for each topic was also calculated for our baseline and the GT documents as well.

#### Classification application

We also present an application of the de-identified data in a realistic machine learning task utilizing the ICD9 (International Classification of Diseases^22^) codes, provided by the MIMIC-III dataset^18^. We implemented a classifier model on the original and de-identified dataset to predict their ICD9 disease code from the underlying EHRs. Both the implementation details of the classification model and ICD9 codes used as labels of the documents are described in Section 5.4 of the supplementary material. The utility here is defined in terms of classification accuracy as we compared the different datasets. Table 5a shows the accuracy for the disease prediction application. The comparison benchmarks are different from BLEU scores and Topic modeling. Previously we compared the matching ratio of the de-identified data to the original data, whereas here we are comparing the accuracy of models trained on the de-identified data. We have three models to compare:

- *Non-sanitized*. Refers to the model trained on the original non-sanitized/raw (with PHI) MIMIC-III data.
- *Baseline*. Our first benchmark is the classification model trained on the data de-identified by the model proposed Dernoncourt et al.^15^ as this is the state-of-the-art de-identification model.
- *Ours*. We also train a model with the data de-identified by our Self-attention model. We then compare its prediction accuracy with the aforementioned benchmark model.

**Table 5:** (a) Comparison of EHR classifications on the de-identified MIMIC-III with 3, 5 and 7 different ICD9 codes (or diseases). Complete list of ICD9 codes can be found in Supplementary Materials (Table S7). (b) Execution time in seconds for RNN and self-attention based model on i2b2 dataset.

**Models**	**P(%)**	**R(%)**	**F1(%)**	**Accuracy**		
				**3**	**5**	**7**
Non-sanitized	–	–	–	91.5	84.1	80.9
Baseline	99.91	99.97	99.94	91.5	85.8	83.4
Ours	99.98	98.79	99.37	91.8	88.1	85.8

Work	i2b2		#Parameters			
	Train	Test				
Baseline^15^	794.36	266.43	3,085,440			
RNN (GRU)	802.02	268.18	3,084,940			
Self-attention	**693**.**38**	**124**.**01**	**110,000,000**			

Bold values indicate heighest values of their respective columns.

### Execution time

Table 5b shows the training and testing time for the i2b2 dataset. We calculated the computation time for the Baseline Dernoncourt et al.^15^ model and our GRU based and the self-attention model. These computations were conducted on the same machine having NVIDIA Titan V GPU (12 GB memory).Note, here the testing time refers to the prediction time for an unknown corpus replicating the real-world use-case and the training time denotes a single epoch. Now, from Table 5b, we can see that the testing time for the self-attention model is significantly lower than the RNN based model. Moreover, even though the attention model has almost 35 times more parameters to train, it was able to train the model in less time than both the RNN based models. An RNN based model looks at the tokens sequentially, whereas the self-attention model looks at the whole sentence at the same time and process it in conjunction. Therefore, the self-attention model can parallelize the token inputs and learn the overall concept of a sentence at the same time.

## Discussion

In this work, we examined GRUs, stacked GRUs, and different permutations of these units to increase the recall for the de-identification task. However, we observed that we need to change the core technique to self-attention to achieve faster execution time and better recall. In the following subsections, we discuss our observations and the implications from the results we presented in the previous section.

### Effect of RNN models

In the i2b2 dataset, all three RNN models have better precision than^15^. Although all of these models have over 95% recall value, they fall behind the benchmark model resulting in the lower F1 score. Our GRU-GRU model has better precision than the single GRU model. However, the GRU model has a better F1 score than both GRU-GRU and LSTM-GRU models as it has much higher recall value than both of these models. As mentioned earlier, this is surprising as the additional RNN should have produced a better result. We speculate that this extra layer led to memorizing more training data, which consequently resulted a poor performance in the test set. For the MIMIC-III dataset, the results are almost similar to the i2b2 dataset where the GRU model has the best F1 score. However, the performance differences between the proposed RNN models are nominal. The better performance of the GRU model signifies that the extra RNN layer does not help improve the performance. Moreover, the stacked RNN models take significant time to converge.

### Effect of attention models

In the i2b2 dataset, our attention model outperforms all the RNN based models. This model is finetuned on a model pretrained on Wikipedia. The large volume of the corpus makes the model better equipped to handle Out-of-Vocabulary words. Also, our results suggest that the context-aware word embedding used for the pretrained model gave an edge during processing different uses of the same word as noun and verb. On the MIMIC-III dataset, the attention model has the highest precision. However, the low recall value indicates the high number of false-negative values. Our analysis of the result shows that most of the errors accrued due to tokenizations errors. For example, a “[”,“*” or“]” was counted as PHI token in the dataset which our self-attention model predicted as not-PHI as the model is not custom-tailored to detect these patterns.

### Effect of CRF

From Table 2a, we observe that for the GRU model, there is a substantial gain in the F1 score when we use CRF instead of the softmax layer. The CRF layer helps the model to consider the correlations between neighboring labels and jointly decode the labels. A softmax function individually predicts the label based on the vectors received from the context modeling layer. It does not take into account the prediction of the neighboring tokens, in contrast to the CRF algorithm. This argument is also applicable to the results found for the self-attention model. The attention model has a 0.823% rise in precision when using the softmax layer. However, the recall value for the attention model increases by 1.01%, resulting in a 0.098% F1 improvement when CRF is used.

### Effect of character embedding and dropouts

From Table 2a, we observed that using the character embedding gave an 8.637% gain in the recall value. This improvement is expected as without the character embedding the model did not have any way of learning the similarity between words. This result motivated us to use the character embedding for all of our other RNN based models. Furthermore, we avoid the overfitting issues by regularization and dropout, employed in all layers of the deep neural network. Our results in Table 2a denote that a 50% dropout resulted in 1.49% improvement compared to the 0% dropout with GRUs. Higher dropout values ($> 50\%$) reduced the probability of overfitting but also reduced the model’s ability to learn. Therefore, we used a maximum of 50% dropout while training the models for results in the *test dataset*. For the self-attention model, the F1 score (for test set) follows a similar pattern. Lower dropout values converge quickly ($<20$ epochs) and overfit the training dataset. However, it performs poorly on the test sets as it only achieves 97.923% recall, which improves for 25% dropouts. Note, for 50% dropout the performance of the self-attention model drops in terms of precision (93.78%) which is essential for utility (details in “Utility analysis” section).

### Effect of precision and recall on the utility

Our model and earlier baseline work from Dernoncourt et al.^15^ did not achieve 100% precision or recall and resulted in higher utility scores. This is entirely due to the number of tokens available or removed in the de-identified documents determined by the FNs or FPs, respectively. For example, our $0.58\%$ recall improvement reduced the number of PHI instances compared to the baseline approach. It is important to note that we have higher precision ($+0.11\%$), which means we did not remove more tokens wrongfully in comparison to the baseline eventually, increasing the BLEU score slightly (0.023). These observations are consistent for $n=\{2,3\}$ grams as we have a better utility score compared to the baseline in all cases. It is critical to understand that BLEU scores do not necessarily represent the absolute utility of the de-identified data. As mentioned earlier, we treated de-identification as a machine translation problem where any non-sanitized (with PHI) document is translated into sanitized (without PHI) documents. The ratio of the matched word in both sanitized and non-sanitized documents thus directly reflects the utility of the sanitized documents when considering BLEU as our utility metrics, as the privacy of the sanitized document is guaranteed with the recall percentage of the de-identification model.

Table 4 shows that our model offered similar (overall) word frequency as the baseline and GT. This result is consistent with our previous utility results in “Utility analysis” section, where the GT has a $100\%$ precision and recall value. Similar to our prior argument, our model has a precision value of $98.031\%$ better than the current baseline^15^. Therefore, more non-sensitive words were left in the de-identified document compared to the baseline. However, we do not perform better than GT in the topic-wise word frequency for all topics. From Table 4, we can see that our model has a better score than the baseline for all the topics except Topic-2. Nevertheless, we compensate for that performance cumulatively on other topics.

The results in Table 5a show that all accuracy values decreases with the increment in the disease numbers. For example, when we consider the first three diseases, the accuracy is 91.8%, which decreases to 88.1% for the first fives in our sanitized dataset. This decreasing accuracy is prevailing across both de-identified datasets and different disease numbers.

In an automated de-identification task, a machine learning model can often simplify it by identifying the less-frequent words. This is possible as PHI tokens (*i.e.*, Names, Location, etc.) may appear only a few times in the whole corpus. Furthermore, they usually follow the same pattern (*i.e.*, Dates). It is noteworthy that these words do not affect the diagnosed disease as they are unimportant while making an ICD9 prediction. As both de-identification tasks essentially reduce the number of tokens from the original MIMIC-III corpus, it inherently simplifies the learning complexity imposed by inconsequential tokens (for disease prediction). Hence, the two models trained with the de-identified datasets from Dernoncourt et al.(Baseline) and ours provide better accuracy than the non-sanitized model.

The model trained with the de-identified dataset from our proposed model has better accuracy than the baseline. A closer look at the predicted results from both de-identification models showed that our self-attention model de-identified $7.91\%$ of the total words, whereas Dernoncourt et al.de-identified $8.19\%$ from the whole corpus. This higher percentage of the de-identified words may suggest better privacy (including FPs); although, our self-attention model achieves a higher precision that establishes the fact that our model de-identified a lower number of non-sensitive words (lower FPs). These non-sensitive words have been attributed to the incremental accuracy improvement compared to the other approach.

### Limitations

In this article, we conducted experiments on three different real-life datasets varying various neural network parameters. Below, we discuss some of our observations and limitations of the proposed approach.

- **Class Imbalance**: We measure performance based on precision and recall (relies on TP, FP, and FN). We did not discuss anything about the true negatives or negative classes in general. One of the interesting sides of the problem is the size of the negative classes (number of non-sensitive tokens) compared to the positive ones. For example, in i2b2 we had only 11,243 positive (PHI) tokens, whereas negative class was 35 times bigger with 394,790 tokens. Currently, we do not incorporate this imbalance in our data processing or architecture model. In the future, we plan to investigate further this issue to increase performance.
- **Preprocessing MIMIC-III**: As mentioned earlier, we manually annotated the MIMIC-III dataset with existing data. This process may contain some edge cases which we could not manually intervene due to the large size of the corpus. Nevertheless, we used the Nursing Note dataset to verify our performance over larger corpus, including the original annotations. Due to the unavailability of a larger corpus with original PHI labels, we think our experiments on the MIMIC-III dataset will provide insights on how the proposed models will perform head-to-head for a large dataset.
- **Quantifiable Privacy and Utility Model**: As discussed in “Utility analysis” section, the privacy loss can be quantified by the recall metric. Since a single false negative token can re-identify an entire EHR at the worst case (i.e., patient’s name), a maximum recall of 100% should ensure its privacy. However, increasing recall might adversely affect the precision (via increasing false positives), which will consequently reduce the utility of the underlying data. Measuring the privacy-utility tradeoff is an interesting future research direction.
- **Transfer Learning**: Although we experimented with different real-life datasets, we did not check the transferability of the model. Theoretically, the model should perform reasonably well in case of transfer learning. However, we could not experiment due to the lack of compatible datasets. This is a possible future work for our research.
- **Inference Attacks**: Like all existing works for this problem, our goal is to satisfy the HIPAA privacy requirements. We do not evaluate the re-identification risk through linkage or inference attacks^4^. Please note that existing privacy models (e.g., differential privacy, k-anonymity) cannot be used to release unstructured textual data. By definition, the release of raw textual data will violate the definition of differential privacy. Indeed, if the goal is to release partial information (e.g., frequent keywords), then it is possible to satisfy privacy definitions like differential privacy. In this paper, our goal is to release the raw textual data without PHI instances. We reiterate that this is a significant problem for data owners. Currently, data owners use costly manual approach. An accurate automated approach will significantly reduce the cost of de-identification. Developing an efficient anonymization algorithm for textual data that can provide a provable privacy guarantee remains an open research challenge.

## Methods

In this section, we discuss our proposed method in detail, which has four major layers with varying components in each layer (except Data Layer). In the following subsections, we describe these layers sequentially as appear in the processing pipeline. Furthermore, our implementation of the models is available at^23^. More detail of the method is added in the supplementary information.

**Figure 1:**
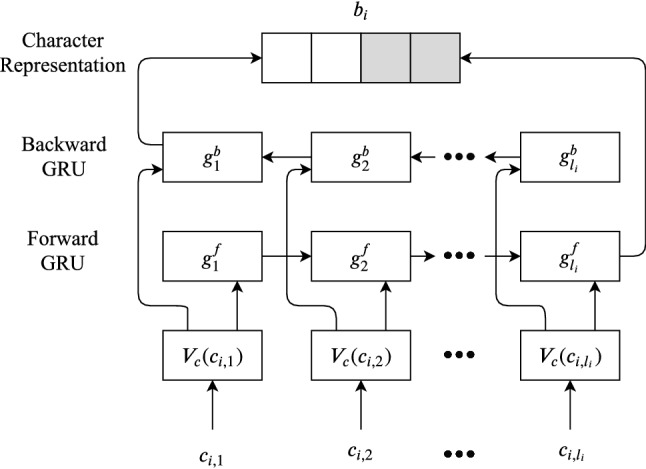
The structure of the Embedding Layer.

### Data layer

The Data Layer preprocesses the input EHRs considered for the network. The data layer splits every sentence of the EHR documents into sequences of tokens (words). Then, each token is assigned to a unique numeric value according to their occurrence sequence in the datasets. In other words, the tokens in the datasets were serialized and each token had a fixed serial number as their identifier. These numbers are then used to convert the sentences into sequences of numeric values or vector. In other words, one numeric vector will represent a sentence of the EHR. The labels (PHI subcategories) in the training set are also indexed using unique numeric values using similar mechanism to the tokens. For attention model, these numeric values are the ids found from the vocabulary list. The numeric representations of the sentences and their associated label sequences are fed into the embedding layer.

### Embedding layer

The embedding layer captures the semantic meaning and features by embedding each token to a vector. Token embeddings improve the semantic information captured by the neural networks in the context modeling layer. However, there are still unperceived information by the network in case of out-of-vocabulary or misspelled infrequent words, a different noun or verb endings. One solution is lemmatizing each token before training the network, but it will also cause the loss of information (e.g. the distinction between noun and verb). To solve this problem, we used the character level embedding for each token in addition to word level embedding.

**Character level embedding.** We used a bidirectional GRU network to determine the character level embedding. In our models, we depart from other benchmark methods, which predominantly used a bidirectional LSTM for computing the character level embedding. A bidirectional GRU consists of a forward and a backward GRU. The hidden states of the forward GRU capture information from the past positions whereas backward units capture information from future positions as it is fed in reverse order. The final hidden state of a backward or forward GRU summarizes the entire character sequence. Figure 1 shows the network structure for such character embedding. Let $s=\{s_1,\ldots ,s_n\}$ be the input sentence with *n* is the length of the sentence. Also let $c_{i,1},\ldots ,c_{i,l_i}$ be the sequence of characters that comprise the $i\hbox{th}$ token $s_i$, where $l_i$ is the number of characters in $s_i$. The character-level token encoder generates the character based token embedding of $s_i$ by first mapping each character $c_{i,j}$ to a vector $V_c(c_{i,j})$, called a character embedding. Then the sequence $V_c(c_{i,1}),\ldots ,V_c(c_{i,l_i})$ is passed to a bidirectional GRU, where $g_1^b,\ldots ,g_{l_i}^b$ and $g_1^f,\ldots ,g_{l_i}^f$ are the hidden states for backward GRU and forward GRU layer respectively. The character level representation $b_i$ is calculated by concatenation of the outputs of hidden state $g_1^b$ and $g_{l_i}^f$.

**Word level embedding.** A general purpose pre-trained word-embedding like Glove^24^ or Word2Vec^25^ is not available from clinical notes. Hence, for the fixed word level embedding, we used the the general purpose GloVe^24^ pretrained model. The publicly available GloVe model contains token embedding for 6 billion unique tokens. We retrieved the vectors corresponding to the EHR tokens with 100 dimensions from GloVe and used them hereafter. The final output $e_i$ from the token embedding layer for $i\hbox{th}$ token $s_i$ is the concatenation of the word embedding $V_T(s_i)$ and the character based token embeddings $b_i$. In summary, when the character embedding layer receives a sequence of tokens $s_{1:n}$ as input, it will output the sequence of token embedding $e_{1:n}$. It is noteworthy that we also used a dynamic embedding scheme where the usage or placement of a token in a sentence determines its numeric values on a fixed size vector.

### Context modeling layer

**Figure 2:**
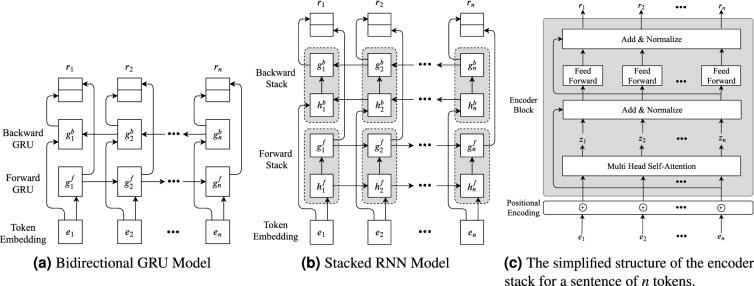
Structure of different context modeling layer with different components.

#### RNN models

The proposed RNN models use both LSTM and GRU units. Each model employs bidirectional RNN sublayer as it helps to capture both future and past information Previous state-of-the-art approach^15^ utilized single bidirectional LSTMs, whereas we use bidirectional GRUs and stacked GRU+LSTM models.

**GRU Model.** As mentioned in the architecture, GRU units offer fewer parameters compared to LSTMs. In our first variant of the context modeling layer, we used a bidirectional GRU layer with one forward GRU and one backward GRU. In contrast to the construction of character-level embedding, the final output is calculated by concatenating the output of hidden states at each time step. Figure 2a depicts the construction of the context modeling layer using such bidirectional GRU. Here, $g^f = \{g^f_1,g^f_2,\ldots ,g^f_n \}$ and $g^b = \{g^b_1,g^b_2,\ldots ,g^b_n \}$ be the hidden states of the forward and backward GRU, respectively. The final output vector $r_i$ for the $i\hbox{th}$ token of a sentence (length *n*) is formed by concatenating the forward and backward hidden states $g^f_i$ and $g^b_i$ respectively (i.e., $r_i=(g^f_i.g^b_i)$). These output vectors $r_i$ will be used by the label decoding layer.

**LSTM-GRU Model.** Then, we analyzed the effect of deeper models by stacking RNN models. For the second variant, we used two bidirectional GRU layers. Let $g^f$ and $g^b$ be the set of hidden states for forward and backward GRU respectively. $g^f_i$ and $g^b_i$ are the hidden states of forward and backward GRU at $i\hbox{th}$ time step. Also, let $h^f$ and $h^b$ be the set of hidden states for forward and backward LSTM, and the hidden states of forward and backward GRU at $i\hbox{th}$ time step be $g^f_i$ and $g^b_i$. For the forward RNN layer, the output of hidden state $h^f_i$ is relayed to $g^f_i$. The backward RNN layer followed the same mechanism. The final output $r_i$ at $i\hbox{th}$ time step for the $i\hbox{th}$ token in a *n* length sequence is formed by concatenating the output of hidden state $g^f_i$ and $g^f_i$ i.e., $r_i=(g^f_i.g^b_i)$. Figure 2b illustrates the construction of this model.

**GRU-GRU Model.** The final variant of the stacked RNN model follows the same structure as the previous model, but the forward and backward LSTM sublayer was replaced with a siamese forward, backward GRU sublayer respectively.

#### Attention model

The RNN models described above are sequential in nature. The hidden states of these models for a time step is a function of the previous step. During training, this sequentiality becomes pivotal while processing longer sentences as memory constraint curtail the opportunity of batch processing. To overcome such constraint, we are using a multi-headed self-attention mechanism for generating a dynamic word embedding instead of the fixed word embedding techniques (detail in Supplementary Information). The dynamic context-aware embedding eliminated the necessity of an additional RNN layer for context analysis. The input for this model is a vector of numeric values incoming from the data layer. The output values generated from this model are forwarded to the Label Decoding Layer.

**Multi-headed self-attention.** The multi-headed self-attention mechanism used here was first introduced by Vaswani et al.^10^ as a replacement for firmly established RNNs for sequence modeling and transduction problems such as language modeling and machine translation. The model introduced in Vaswani et al.^10^ namely, Transformer follows the encoder-decoder architecture of traditional transduction models. Instead of using an RNN or CNN, Transformer uses only attention mechanisms. However, we used a bidirectional Transformer, which is often referred to as Transformer Encoder. This is similar to the BERT^11^, which is conditioned on both the left and right context.

Self-attention sublayers employ *h* attention heads. The sublayer output is retrieved from concatenating the results from each head and a parameterized linear transformation. Each attention head operates on an input sequence passed from the data layer. This input vector is added with positional encoding and forwarded to the self-attention sublayer. Figure 2c shows a simplified structure of the mechanism where the self-attention sublayer receives input sequence $e=e_1,\ldots ,e_n$ of *n* tokens after positional encoding and computes a new sequence $z = z_1,\ldots ,z_n$ of the same length. Each output element, $z_i$, is computed as the weighted sum of linearly transformed input elements: $z_i = \sum _{j=1}^{n}\gamma _{ij}(e_{ij}W^V)$. Each weight matrix, $\gamma _{ij}$, is computed using softmax function: $\gamma _{ij} = \frac{\exp a_{ij}}{\sum _{k=1}^n \exp a_{ik}}$. A compatibility function is used to compute $a_{ij} = \frac{(e_iW^Q)(e_jW^K)^T}{\sqrt{d_z}}$,which compare two input elements. This scaled dot product was chosen as the compatibility function due to its computational efficiency. $d_z$ used for computing $a_{ij}$, is the dimension of the matrix $e_jW^K$ and $W^Q, W^K, W^V$ are parameter matrices. These parameter matrices are different for each layer and attention head and learned during training.

### Label decoding layer

The label decoding layer takes vectors containing the context information as input and predicts the PHI instances (sub-categories like DOCTOR, PATIENT, HOSTPITAL, etc. shown in Table S3 in the supplementary materials). As PHI instances could have multiple tokens/names we used the BIOES (which stands for Begin, Inside, Outside, End and ,Single, indicating the position of the token in the PHI instance) tagging scheme^26^, which distinguishes between the end of a multi-token PHI instance and single token PHI instance. Now, we are using two different methods–softmax and CRF model for predicting labels from the vectors calculated in the context modeling layer. For the RNN models, we used an Fully Connected Neural Network (FNN) before the softmax/CRF layer. However, as the attention model uses an FNN within the encoder block, we directly used the output of the attention model as an input to the softmax/CRF layer.

**Softmax Layer.** The softmax layer normalizes the values received from the FNN to probabilities. Let’s assume, this layer receives $r=\{r_1,\ldots ,r_n\}$, as the input sequence where $r_i$ is the $i\hbox{th}$ token’s score. Then, the probability for the $j\hbox{th}$ label (PHI) for $i\hbox{th}$ token would be, $q_j(r_i) = \frac{\exp {r_{ij}}}{\sum _k\exp {r_{ik}}}$. Here *k* is the total number of labels. The network is trained to minimize the cross-entropy loss, ${\mathcal {L}}(p,q)$ where *p* is the original probability for $j\hbox{th}$ label.

**CRF Layer.** CRF models consider the correlations between labels in neighboring positions and jointly decode the best chain of labels for a given input sentence. The use of CRF algorithm to predict the labels from the features extracted in the context modeling layer is a standard practice in a NER task. Our experimental results further emphasized the effectiveness of the CRF algorithm while predicting interdependent labels. Let, $y=\{y_1,\ldots ,y_n\}$ represent a generic sequence of labels for *r*. $\theta (r)$ denotes the set of possible label sequences for *r*. The probabilistic model of CRF determines a conditional probability, *p*(*y*|*r*; *W*, *b*) over all possible label sequences *y* with respect to *r*, where *W* and *b* are the weight vector and bias. During training, we used the maximum conditional likelihood estimation. For a training set, $\{(r_i,y_i)\}$, the logarithm of the likelihood is given by: ${\mathcal {L}}(W,b) = \sum _{i}\log {p(y|r;W,b)}$. Maximum likelihood training chooses parameters such that the log-likelihood ${\mathcal {L}}(W,b)$ is maximized. Decoding is to search for the label sequence $y*$ with the highest conditional probability. i.e.$y* = {{\,\mathrm{\mathrm{argmax}}\,}}_{y\epsilon \theta (r)}{p(y|r;W,b)}$.

### Loss function

In addition to the log-likelihood and cross-entropy loss calculation, we modified the loss function to favor recall over precision. We introduced a new hyperparameter, $\delta _{p}$, which dictates the maximum weight of the penalizing factor, $\rho$. This penalizing factor ($\rho$) depends on the false negative rate and $\delta _{p}$. The false negative rate, *FNR*, is defined as $FNR = FN/(1+FN+TP)$, where *FN* and *TP* are the count of false negatives and true positives, respectively. Notably, $\rho$ is calculated using the equation, $\rho = FNR\times \left[ (\delta _{p}-1)+1\right]$. We formulated the eq. of *FNR* and $\rho$ in a way which ensures that the *FNR* is never $\infty$ and $\rho$ always has a value within $[0,\delta _{p}]$. Finally, the recall heavy loss is the original loss $L_{regular}$ weighted by $\rho$ ($L_{recall} = L_{regular}\times \rho$).

## Conclusion

We proposed an architecture employing new deep learning methods to de-identify textual data and analyzed their performance with existing methods. Experimental results showed that our self-attention based approach is computationally efficient and performs better than the state-of-the-art models. We also introduce novel approaches to measure the utility of the de-identified documents and analyze the relationship between utility, precision and recall value of neural network-based models. Finally, our proposed loss function improves the recall value compared to regular loss value, although there is a trade-off between the recall and precision values. In the future, we would like to find the interoperability of these models on different datasets and perform transfer learning. We also plan to measure the re-identification risks of our proposed method.

## Supplementary information

Supplementary material 1
