# Supplementary material for: De-identification of electronic health record using neural network
Source: Sci Rep. 2020 Oct 29;10:18600. doi: 10.1038/s41598-020-75544-1 (PMC7596089; doi:10.1038/s41598-020-75544-1)
Supplement: Supplementary file 1 — Supplementary material 1 [file 41598_2020_75544_MOESM1_ESM.pdf]

De-identification of Electronic Health Record using Neural Network

Tanbir Ahmed[1,*], Md Momin Al Aziz[1], and Noman Mohammed[1]

[1]University of Manitoba, Department of Computer Science, Winnipeg,Canada
[*]tahmed@cs.umanitoba.ca

1 Architecture Detail

Our proposed architecture consists of four primary components as we considered different techniques as building blocks. Figure S1 depicts an overview of the architecture and its different derivations.

1.1 Data Layer

The Data Layer preprocesses the input EHRs (or documents) considered for the network. We used the patient notes and discharge summaries from the i2b2 2014 dataset [1] and the MIMIC-III Critical Care Database [2], respectively. Each document in these datasets corresponds to one patient visit. These tokens in these EHRs are labeled into 18 subcategories as shown in Table S3. Now these subcategories are categorized in to 7 larger categories as outlined by the HIPPA. However, all the proposed models are trained to detect the subcategories. We also used the notes from the Nursing Note [3] dataset, which is also categorized as described above.

The data layer splits every sentence of the documents into sequences of tokens (words). Then, each token is assigned to a unique numeric value. These numbers are then used to convert the sentences into sequences of numeric values or vector. In other words, one numeric vector will represent a sentence of the EHR. The labels in the training set are also indexed using unique numeric values. For attention model, these numeric values are the ids found from the vocabulary list. The numeric representations of the sentences and their associated label sequences are fed into the embedding layer.

1.2 Embedding Layer

The Embedding Layer takes the sequence of tokens from the data layer and generates a unique numeric encoding for each token. Earlier, NLP systems encoded each token as a discrete atomic symbol. These encodings were random and perceived no useful information pertaining to the relationship between two different tokens. Furthermore, it did not consider the token's context in which it was used. Consequently, the neural network trained on these random values could leverage very little information while processing each token sequentially. The unique and arbitrary encoding for each token led to data sparsity, which demanded more data for training neural networks and therefore we do not consider them in our proposed architecture.

Shortcomings of unique encoding are mostly resolved in the Vector Space Models (VSMs) [4], where each token is represented in a continuous vector space. In VSMs, semantically similar words or tokens are mapped to nearby points in a fixed dimensional geometric space. This mapping is called word embedding and are popular in NLP pipelines, where a fixed-sized vector represents one word. These numeric word embeddings showed that the operations among 'King' - 'Man' + 'Queen' is 'Woman'. In our proposed models, we used two different types of embeddings:

Fixed Embedding. In a fixed valued word embedding, each token will be represented with one numeric vector. This vector will remain the same regardless of the context or where the tokens occur in a sentence. For example, the word "right" will always have one embedding even if we had a sentence like "You were right when we made the right turn", where the word right has different meanings depending on the usage. Fixed embeddings are trained via neural networks on larger text corpus like Wikipedia or newspapers, and we used this embedding in the RNN models.

Dynamic Embedding. In the dynamic embedding, tokens have different embeddings depending on their meanings and the order of occurrence in a sentence. For instance, for the same example above, "You were right when we made the right turn"; this embedding will generate different vectors for the two occurrences of "right". The first "right", for instance, would be closer to a word like correct, whereas the second one would denote direction. The dynamic embedding of words could be generated using a bidirectional LSTM or self-attention mechanisms. For the simplicity of the model architecture, we used the latter one in our attention model.

1.3 Context Modeling Layer

From the embedding layer, the token embeddings are passed down to the Context Modeling Layer. This layer captures the information required to understand the relationship between two subsequent tokens. This relationship is referred to as

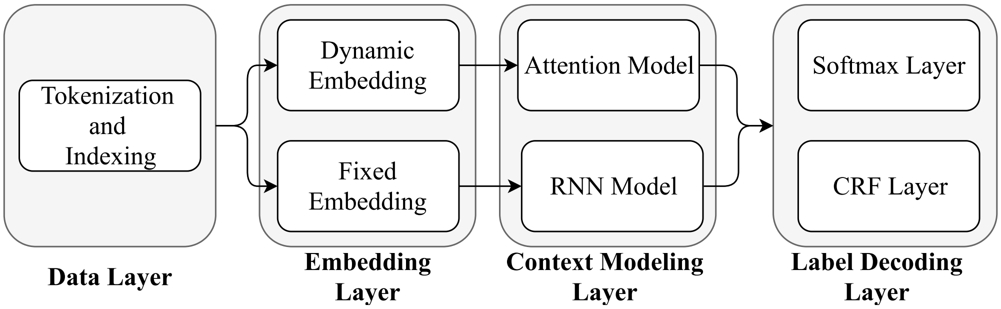

Figure S1. Architecture overview of our proposed de-identification system considering different components

the context of those tokens. The context information is then forwarded to the next layer that determines the label of each token. Notably, traditional (Feed Forward) neural network processes each token embedding individually. It does not have any information about previously processed tokens. Thus, the Feed Forward Network (FNN) fails to take the context information into account.

In contrast to FNN, RNN not only takes the current token as an input example but also takes the previous time steps into account [5]. Therefore, RNN is more suitable for processing sequential data like sentences (the stream of tokens) or documents. RNN correlates information between two sequential time steps through multiplication. Here, information passes through many stages of multiplications, while RNN tries to establish a correlation between the final output and remote input. This makes gradients susceptible to vanishing or exploding. Although in theory RNNs are capable of capturing long term dependencies, due to this gradient vanishing/exploding problem, they fail to do so. LSTMs are a variant of RNN, which solves this issue by introducing a memory unit in the single RNN cell in addition to the hidden states [6]. On the other hand, GRU cells, another variant of RNN is computationally more efficient than LSTMs as they possess a less complex structure. The GRU controls the flow of information like the LSTM, but without having to use a memory cell [7]. Therefore, for capturing both the context information from both sides, we are using a bidirectional GRU structure. We give a brief description of the different variants of RNN (LSTM & GRU) in Supplementary Section 3. We encourage readers to go through this section for a clear understanding of how the RNN variants learn the context.

1.4 Label Decoding Layer

Label Decoding Layer takes the vector containing the context information from RNNs and predicts the appropriate label for the underlying token. We are using a simple feed-forward network, which assigns a score to all PHI labels (`NAME`, `ADDRESS`, `NOT SENSITIVE`, etc.) for each token. These scores represent their likelihood of being the correct label for that token. We considered two different approaches to predict these likelihood values for the context layer.

First, we use the naive approach to normalize these scores into probabilities using a softmax function. The predicted label for each token will be the one with the highest probability.

Second, we use a Conditional Random Field (CRF) [8] model. In a sequence labeling task, it is advantageous to consider adjoining labels as these labels are interdependent. For example, the PHI instance `PATIENT` or `DOCTOR` cannot be followed by the `ORGANIZATION` or `HOSPITAL` instance. Again `STATE` is most likely to be followed by `CITY`. The naive approach is ill-equipped to leverage this correlation between neighboring labels. However, multivariate discriminative models like CRF are capable of utilizing the correlation. CRF determines the probability of a label for a token considering the total input sequence.

2 Related Works

Several architectures have been proposed to de-identify text data in the healthcare domain. Here, we characterized these systems into three major categories: rule-based, machine learning based, and hybrid systems. Below we briefly describe these methods.

2.1 Rule-based Approach

Early machine-assisted de-identification systems were mostly rule-based for text data. These systems used patterns, regular expressions, and dictionary lookups to de-identify different EHRs such as patient notes, discharge summaries, and nursing notes. In one of the earliest attempts in 2005, Douglass *et al.* [13] developed a semi-automated method to allow clinicians to highlight PHI instances on the screen of a tablet PC. Their method could compare and combine the selections of different experts reading the same notes. Another approach from Douglass *et al.* [14] used lexical look-up tables, regular expressions, and simple heuristics to locate PHI instances. It is noteworthy that these rules or fixed dictionary entries were done manually and did not scale for large text corpus containing unstructured healthcare information.

Table S1. Precision (%) and Recall (%) comparison for i2b2 dataset [1].

Methods	Year	Approach	i2b2 dataset [1]	
			Precision	Recall
Liu *et al.* [9]	2015	Token CRF	85.89	82.55
		Character CRF	93.88	88.85
		Rule	28.3	1.742
		Ensemble	92.82	90.91
Dernoncourt *et al.* [10]	2016	ANN+CRF	97.92	97.88
Liu *et al.* [11]	2017	CRF	95.16	91.12
		LSTM	95.26	93.34
		LSTM-Fea	95.43	93.61
		Ensemble	96.46	93.80
		Overall	96.46	93.80
Khin *et al.* [12]	2018	ELMO+LSTM+CRF	98.30	97.37
Our methods	2019	GRU	**98.75**	95.86
		Stacked GRU	**99.01**	95.12
		LSTM-GRU	98.75	95.28
		Modified Loss	98.80	**97.51**
		Self-attention	**98.03**	98.41

2.2 Machine Learning Approach

In recent years, a few machine learning approaches have been proposed as well, which automatically learns the sensitivity of a given text or word sequence. These machine learning algorithms for de-identification mostly include decision trees, support vector machines, hidden markov models, and conditional random fields (CRFs). Szarvas *et al.* [15] proposed an iterative anonymization method on semi-structured documents. Their proposed method labeled all PHI instances inferred from the structure of the document and then utilizes this information to find further PHI instances. In 2014, this area of research received much-deserved attention due to the UTHealth i2b2 de-identification challenge. It was this competition that produced several machine learning based automated tools on the i2b2 2014 dataset.

During this challenge, Chen *et al.* [16] introduced a non-parametric Bayesian hidden markov model using a Dirichlet process (HMM-DP). They managed to score a 91% F1 score (with 87.9% recall) in the competition. Later they improved the HMM-DP model and aligned it with a CRF model, and obtained a 93.7% F1 score (Recall: 91%). On the same competition, He *et al.* [17] proposed a CRF based de-identification system with a significant focus on preprocessing and feature generation from medical records, and achieved 95.71% precision and 90.51% recall, respectively.

2.3 Hybrid Approach

The UTHeath i2b2 de-identification challenge has also produced some hybrid systems where different de-identification pipeline worked individually, and their results were combined with an ensembling algorithm to produce the eventual results. Liu *et al.* [9] proposed a system that uses a token-level CRF, a character-level CRF, and a rule-based subsystem as their de-identification pipelines. They achieved 91.85% F1 score (Precision: 92.82%, Recall: 90.91%) for their ensemble model. Dehghan *et al.* [18] combined the results of a dictionary and rule-based subsystem with a CRF based subsystem. They trained a separate CRF model for each of the PHI types and post-processed the results with hand-crafted rules. On their second submission in the competition, they scored a precision of 96.55% and recall of 93.16%. Yang *et al.* [19] proposed a similar approach. However, they used the CRF model only for the PHI categories with sufficient amount of available training data and keyword spotting and rule-based approach for the PHI categories for fewer sample instances. With a 93.6% F1 score Yang *et al.* [19] was the winner of the de-identification challenge.

In the early stages of deep learning, the hybrid approaches dominated this domain. In 2016, Dernoncourt *et al.* [10] proposed the first neural network for de-identifying patient notes. They used both character and word-level embedding vectors. For character-level embedding, they used a bidirectional LSTM and combined the result with word-level one. Another bidirectional LSTM was used for the context encoding, and finally, they used a CRF for optimizing the label prediction. Until now, this model had the best result in the i2b2 2014 dataset. Therefore, we used this model as our benchmark and compared all our results against it. Yadav [20] *et al.* compared two different variants of RNN (Jordan-type and Elman-type Networks) for the context analysis. They achieved 89.63% and 90.18% F1 for Elman-type and Jordan-type networks respectively. As they did not use the total i2b2 2014 dataset, their results cannot be directly compared with other systems. In 2017, Liu *et al.* [11] proposed another hybrid system with four subsystems based on bidirectional LSTM, CRF and rule-based. Khin *et al.* [12] used a deep

contextualized word-level embedding and bidirectional LSTM character-level embedding together. For other components of the network, they used a bidirectional LSTM and CRF identical to Dernoncourt *et al.* [10]. Table S1 gives a comparison of precision and recall for these hybrid approaches. Experimental results show that our proposed methods are better than all of these attempts.

3 Recurrent Neural Network

RNN is more suitable for processing sequential data like sentence or documents. RNNs preserve the sequential information in hidden states, which cascades many time steps forward effecting the output at each time step. Thus, RNNs create long term dependencies between events separated by multiple time steps. Let's assume that at time step t an RNN cell takes token x_t and information about the previous time step h_{t-1} as input. It preserves the information learned in time step t in hidden state h_t and moves forward this information to the next time step. At time step t, output y_t is as follows:

$$h_t = \tanh(W_h \begin{pmatrix} h_{t-1} \\ x_t \end{pmatrix})$$
$$y_t = W_y h_t$$

Here, W_h and W_y are the weight matrices for the RNN unit. These weight matrices are updated by a learning rule. The change in the values represents the change in the error of the network and is determined during backpropagation by calculating the partial derivative of the error with regard to the weight matrices. This partial derivative is called *gradient*.

3.1 Long Short Term Memory

RNN correlates information between two sequential time steps through multiplication. Here, information passes through many stages of multiplications while RNN tries to establish a correlation between the final output and remote input. This makes gradients susceptible to vanishing or exploding. Although in theory RNNs are capable of capturing long term dependencies, due to this gradient vanishing/exploding problem they fail to do so.

LSTMs are a variant of RNN, which solve this issue by introducing a memory unit in the single RNN cell in addition to the hidden states [6]. An LSTM cell also has four differentiable gates implemented with element-wise multiplication by the sigmoid function. These gates determine the information flow in the memory unit and to the next time step. Let's consider a single LSTM cell that takes x_t, h_{t-1} and c_{t-1}, and generates hidden state h_t, memory unit c_t at t step as follows:

$$\begin{pmatrix} i \\ f \\ o \\ g \end{pmatrix} = \begin{pmatrix} \sigma \\ \sigma \\ \sigma \\ \tanh \end{pmatrix} W \begin{pmatrix} h_{t-1} \\ x_t \end{pmatrix}$$
$$c_t = f \odot c_{t-1} + i \odot g$$
$$h_t = o \odot \tanh(c_t)$$

Here, i, f, o, g represent the gates used in a LSTM cell. i and f determine whether to write or erase information from the memory cell, respectively. On the other hand, the g gate determines how much information should be written in the memory cell, and o decides the amount of saved information sent as output. These decisions are regulated by the weight matrices W and learned during training.

Symbols σ and tanh refer to element-wise sigmoid and hyperbolic tangent functions, and $\odot$ is an element-wise multiplication. The sigmoid function keeps the values within a certain range forcing the gates to behave like a logic gate. This gating mechanism eliminates the matrix multiplication during backpropagation. As evident from the equation, backpropagation from c_t to c_{t-1} only requires element-wise multiplication.

3.2 Gated Recurrent Unit

As shown in Figure S2, the GRU controls the flow of information like the LSTM, but without having to use a memory cell [7]. It just exposes the full hidden content without any control. GRU cells are computationally more efficient as they possess a less complex structure. For capturing both the context information from both sides, we are using a bidirectional GRU structure.

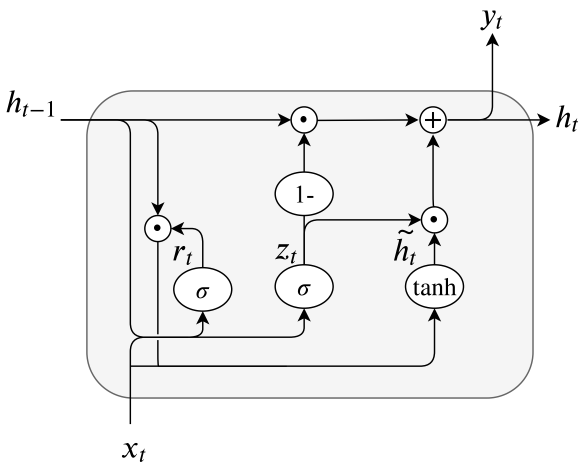

Figure S2. Example of a GRU Cell with $\sigma, tanh$ activation functions and x_t, y_t as the input and output, respectively. h_{t-1} is the hidden state from previous time step and the output is forwarded to the next time step as h_t.

Let's assume a GRU unit takes x_t and h_{t-1} and produces the hidden state h_t using the following formulas:

$$\begin{pmatrix} r_t \\ z_t \end{pmatrix} = \begin{pmatrix} \sigma \\ \sigma \end{pmatrix} W \begin{pmatrix} x_t \\ h_{t-1} \end{pmatrix}$$

$$p_t = r \odot h_{t-1}$$

$$\tilde{h_t} = \tanh(W \begin{pmatrix} x_t \\ p_t \end{pmatrix})$$

$$h_t = z_t \odot h_{t-1} + (1 - z_t) \odot \tilde{h_t}$$

Although GRU does not have any memory unit or output gate, it has a reset and update gate, which are represented with r and z, respectively. Symbols σ, tanh and $\odot$ refer to the same functions as previously mentioned. We utilize GRU and LSTM units in our context modeling layer on different configurations. There are many other variants of RNNs available, but they do not add any meaningful improvement over our performance; hence, we do not report those results.

4 Model Setup

4.1 Parameter Initialization

- **Word Embeddings.** For RNN models, we used Stanford's publicly available GloVe [21] 100-dimensional embeddings, which are trained on 6 billion words from Wikipedia. For the attention model, we used the WordPiece [22] embeddings with a vocabulary size of 30,000.

- **Character Embeddings.** We considered only the lower case characters, and the dimensions are set to 50 as we randomly initialize them.

- **Weight Matrices and Bias Vectors.** The other trainable matrices and vectors are initialized using Xavier Initialization [23]. Bias vectors are initialized to zero except the forget gate bias in the bidirectional RNN layers (initialized to 1.0).

4.2 Optimization Algorithm

All of the models were trained using Adam [24] optimizer without any decay rate tuning, and updating all the parameters at each gradient step. We choose an initial learning rate of $1e-5$ for all of the models. We used a gradient clipping of 5.0 to reduce the effect of 'gradient exploding' as well. We explored with stochastic gradient descent, but it did not add any meaningful improvement upon the Adam optimizer in our primary experiments. For regularization, in RNN models, a dropout was applied to the embedding and context modeling layer before forwarding to the label decoding layer. In the attention model, a dropout was used only before the label decoding layer. For 100 epochs, the RNN model takes around 33 hours to train on Titan V (12GB) graphics processing unit for the i2b2 dataset. For the same configuration, these model takes around 24 hours to

Table S2. Training Hyperparameters.

Layer	Hyper-parameter	Value
Embedding	GRU hidden state dimension	25
	Word embedding dimension	100
Context Modeling	GRU hidden state dimension	150
	LSTM hidden state dimension	150
Attention	Attention Head Number	12
	Attention Block	12
	Hidden Size	768
Dropout	Dropout rate	0.5
	Initial learning rate	1e-5
	Gradient clipping	5

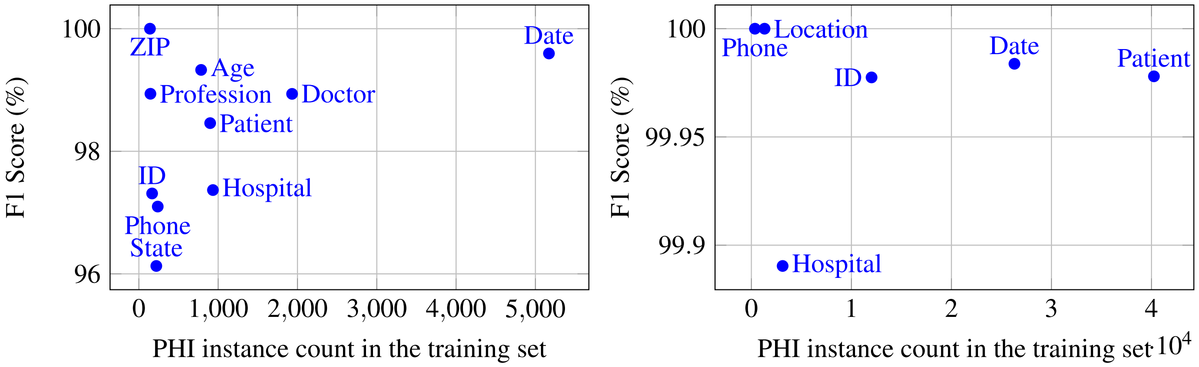

(a) i2b2 Dataset

(b) MIMIC-III Dataset

Figure S3. Impact of the number of PHI instances in the training set on the attention model's performance for each PHI category in the i2b2 2014 and MIMIC-III dataset.

train on the MIMIC-III dataset [2]. The attention model takes around 15 hours and 12 hours on the i2b2 2014 and MIMIC-III datasets, respectively.

Early Stopping. We used early stopping based on validation set performance. The model automatically stops training if the accuracy did not improve for 10 consecutive epochs.

Fine Tuning. For each of the embeddings, we fine-tuned initial embeddings, modifying them during gradient updates of the neural network model by back-propagating gradients. The effectiveness of this method has been previously explored in sequential and structured prediction problems [25].

Dropout training. We applied different dropouts to reduce overfitting and regularize the underlying models. The dropouts are applied succeeding the embedding layer and the attention layer, and before the CRF layer.

4.3 Hyperparameter Tuning

Table S2 gives an overview of the hyperparameters we analyzed. We tuned these hyperparameters on the validation sets by random search. Due to page constraints, we report only the significant ones. For example, the most important character embedding hidden state dimension, attention head, and encoder block were set as 25, 12, and 12, respectively, whereas the hidden size for the attention model was 768.

5 Additional Results

5.1 Dataset Details

Table S3 shows the details for the i2b2 2014 [1] and MIMIC-III [2] datasets.

5.2 Category-wise Precision and Recall

Table S4 presents the category-wise precision, recall and F1 scores listed in Table S3. For the i2b2 dataset, the `DATE` and `AGE` category have the highest recall value, although both have lower precision than the `ID` category, which has the highest

Table S3. PHI token count in each category for i2b2 2014 and MIMIC-III dataset.

PHI Instances		i2b2 2014 [1]		MIMIC-III [2]	
**Category**	**Sub-Category**	**Train**	**Test**	**Train**	**Test**
DATE	NA	7450	4931	30843	6625
NAME	DOCTOR, PATIENT, USERNAME	4425	2833	54084	14525
AGE	NA	1254	774	36	0
CONTACT	PHONE, FAX, EMAIL	381	229	379	75
ID	MEDICALRECORD, IDNUM, DEVICE, BIOID	910	639	12665	3257
LOCATION	HOSPITAL, CITY, STATE, STREET, ZIP, COUNTRY, ORGANIZATION, LOCATION-OTHER	2306	1680	7423	1819
PROFESSION	NA	263	157	-	-
**Total**	**NA**	**16962**	**11243**	**105430**	**26301**

Table S4. HIPAA defined PHI category wise Precision and recall comparison for the self-attention model for the i2b2 2014 and MIMIC-III dataset.

HIPAA PHI	i2b2 2014			MIMIC-III		
	**P**	**R**	**F1**	**P**	**R**	**F1**
ID	99.185	96.358	97.751	100.00	99.955	99.978
Contact	99.113	95.717	97.386	100.00	100.00	100.00
Name	98.929	98.456	98.692	99.959	99.997	99.978
Location	94.832	96.122	95.473	99.876	100.00	99.938
Date	98.840	100.00	99.416	99.968	100.00	99.984
Age	98.206	99.176	98.689	-	-	-
Profession	91.554	98.905	95.088	-	-	-

precision value. The lowest F1 score is for the `CONTACT` type. For MIMIC-III dataset, the lowest F1 score was found for the `LOCATION` category, which is similar to the result for the i2b2 dataset as the location names were sparse. The MIMIC-III dataset did not have any `AGE` or `PROFESSION` type of PHI is the test set. Hence, the respective columns are left blank.

5.3 Effect of training set size

Figure S3 depicts the impact of the number of PHI instances in the training set on the attention model's performance. The horizontal axis of Figure S3 represents the total number for each category of PHI instances in the training set. The vertical axis shows the F1 score for each of these categories in the test set. As expected, entities with a large number of labeled instances in the training dataset tend to be detected more accurately. However, the correlation is far from conclusive. Some instances with a lower number of labeled instances are detected more accurately than some types with a higher number of labeled instances. This indicates that some instances are harder to detect than others. For example in the i2b2 dataset (Figure S3a), although `ZIP` type PHI has fewer labeled data than `DOCTOR` or `HOSPITAL`, it is more accurately detected than the latter ones. This result is expected since tokens containing a `ZIP` number are typically similar, while names of hospitals and doctors can appear in different forms.

5.4 EHR Classification Application

As the BLEU scores for two different corpus provide a deterministic value, it can act as the general-purpose utility metric. Here, we used the de-identified data in a real-life application (Disease Prediction) to analyze a more task-specific utility.

Table S5. Selected Diseases and their ICD9 for EHR classification task

#	Disease Description	ICD9 Codes	EHR No.
1	Subendocardial infarction, initial episode of care	410.71	307
2	Acute respiratory failure	518.81	471
3	Congestive heart failure, unspecified	428.0	387
4	Coronary atherosclerosis of native coronary artery	414.01	545
5	Pneumonitis due to inhalation of food or vomitus	507.0	287
6	Pneumonia, organism unspecified	486	274
7	Intracerebral hemorrhage	431	126

Table S6. Comparison of EHR classifications on the de-identified MIMIC-III with 3, 5 and 7 different ICD9 codes (or diseases). Complete list of ICD9 codes can be found in (Table S7)

	P(%)	R(%)	F1(%)	Accuracy		
				3	5	7
Non-sanitized	-	-	-	91.5	84.1	80.9
Baseline	99.91	99.97	99.94	91.5	85.8	83.4
Ours	99.98	98.79	99.37	91.8	88.1	85.8

Task Description. We used a supervised deep learning model for EHR classification task to measure the utility of the de-identified data. Each EHRs in the MIMIC-III dataset [2] is associated with multiple International Classification of Diseases (ICD9) [26] code. We propose a classifier model on the MIMIC-III dataset (both original, and de-identified) to predict these ICD9 disease code from the underlying texts. Like the analysis of the BLEU score and topic modeling, we have three models to compare:

- *Non-sanitized*. Refers to the model trained on the original non-sanitized/raw (with PHI) MIMIC-III data.
- *Baseline*. Our first benchmark is the classification model trained on the data de-identified by the model proposed Dernoncourt *et al.* [10] as this is the state-of-the-art de-identification model.
- *Ours*. We also train a model with the data de-identified by our Self-attention model. We then compare its prediction accuracy with the aforementioned benchmark model.

Data Selection. As previously mentioned, each document in the MIMIC-III dataset is associated with multiple ICD9 codes. Now, for our de-identification task, we randomly selected 4,441 documents. Now, we labeled this dataset with their associated ICD9 codes. Table S5 shows the ICD9 codes we selected and the number of the documents related to these codes. We selected seven ICD9 codes that have the highest frequency in the original dataset. Thus, the classifier's training dataset is a subset of the original MIMIC-III dataset that we used for the de-identification task. Furthermore, each document is associated with multiple ICD9 codes. So, there are overlaps of documents between the EHR numbers as shown in Table S5.

Classification Model. We used the same model architecture for the classification task as the model used in our de-identification task with two major distinctions. First, we modified the 'Data Layer' (Sec 1.1) to consider each document as one sequence. Thus, the maximum sequence length used is the highest word/token count for any document in the dataset. Second, we changed the 'Label Decoding Layer' (Sec. 1.4) to predict the ICD9 codes of the documents, instead of predicting the label (PHI categories) for each token of the documents. We used only a softmax layer after receiving the scores from the FNN layer. The softmax layer predicted the probabilities for each of the ICD9 codes for the input documents. In keeping similarities with our previous discussion of the softmax layer, let's assume, this layer receives $r = \{r_1, \ldots, r_n\}$, as the scores for each of the ICD9 codes. Then, the probability for the jth ICD9 code for the input document would be, $q_j(r_i) = \frac{\exp r_{ij}}{\sum_k \exp r_{ik}}$. Here k is the total number of ICD9 codes. We considered $k = \{3, 5, 7\}$ for our experiments. For each dataset, we trained three models to distinguish between three, five, and seven categories (ICD9 Codes) of documents.

Results and Discussions. Table S6 shows the accuracy for the disease prediction application. As mentioned previously, we trained the models to predict three different numbers of ICD9 codes for each dataset. The disease codes were selected sequentially From Table S5, as the first three diseases were included for the three disease prediction. These diseases were also present in five and seven disease classifiers.

Table S7. ICD9 codes considered in 3, 5 and 7 disease classifiers where the codes are described in Table S5

Classifier	410.71	518.81	428.0	414.01	507.0	486	431
3	✓	✓	✓	×	×	×	×
5	✓	✓	✓	✓	✓	×	×
7	✓	✓	✓	✓	✓	✓	✓

The experimental results in Table S6 demonstrate that all accuracy values decreases with the increment in the number of diseases. For example, when we consider the first three diseases (from Table S5), the accuracy is 91.8%, which decreases to 88.1% for the first fives in our sanitized dataset. This decreasing accuracy relation is prevailing across both de-identified datasets and a different number of diseases.

In an automated de-identification task, a machine learning model can often simplify it by identifying the less-frequent words. This is possible as PHI tokens (*i.e.*, Names, Location, etc.) may appear only a few times in the whole corpus. Furthermore, they usually follow the same pattern (*i.e.*, Dates). It is noteworthy that these words do not affect the diagnosed disease as they are unimportant while making an ICD9 prediction. As both de-identification tasks essentially reduce the number of tokens from the original MIMIC-III corpus, it inherently simplifies the learning complexity imposed by inconsequential tokens (for disease prediction). Hence, the two models trained with the de-identified datasets from Dernoncourt *et al.* (Baseline) and ours provide better accuracy than the non-sanitized model.

Now, the model trained with the de-identified dataset from our de-identification model has better accuracy than the baseline. A closer look at the predicted results from both the de-identification model showed that our self-attention model de-identified 7.91% of the total words in the dataset, whereas Dernoncourt *et al.* de-identified 8.19%. The higher percentage of the de-identified words may suggest better privacy; however, our self-attention model has a higher precision value than Dernoncourt *et al.* , which signifies that our model de-identified a lower number of non-sensitive words. These non-sensitive words helped our classification model achieve better accuracy than the baseline.

References

1. Stubbs, A., Kotfila *et al.* Automated systems for de-identification of longitudinal clinical narratives. *JBI'15* (2015).

2. Gehrmann, S., Dernoncourt, F., Li, Y., Carlson, E. T. *et al.* Comparing deep learning and concept extraction based methods for patient phenotyping from clinical narratives. *PLOS ONE* (2018).

3. Lee, J., Scott *et al.* Open-access mimic-ii database for intensive care research. In *2011 Annual International Conference of the IEEE Engineering in Medicine and Biology Society* (IEEE, 2011).

4. Nematzadeh, A., Meylan, S. C. & Griffiths, T. L. Evaluating vector-space models of word representation, or, the unreasonable effectiveness of counting words near other words. In *CogSci* (2017).

5. Goller, C. & Kuchler, A. Learning task-dependent distributed representations by backpropagation through structure. In *ICNN* (1996).

6. Hochreiter, S. & Schmidhuber, J. Long short-term memory. *Neural computation* (1997).

7. Bahdanau, D., Cho, K. & Bengio, Y. Neural machine translation by jointly learning to align and translate. *arXiv:1409.0473* (2014).

8. Lafferty, J., McCallum *et al.* Conditional random fields: Probabilistic models for segmenting and labeling sequence data. (2001).

9. Liu, Z., Chen *et al.* Automatic de-identification of electronic medical records using token-level and character-level conditional random fields. *In JBI* (2015).

10. Dernoncourt, F., Lee, J. Y., Uzuner, O. & Szolovits, P. De-identification of patient notes with recurrent neural networks. *In JAMIA* (2016).

11. Liu, Z., Tang, B. & Wang, X. De-identification of clinical notes via recurrent neural network and conditional random field. *In JBI* (2017).

12. Khin, K., Burckhardt *et al.* A deep learning architecture for de-identification of patient notes: Implementation and evaluation. *arXiv preprint arXiv:1810.01570* (2018).

13. Douglass, M., Clifford, G. D. *et al.* Computer-assisted de-identification of free text in the mimic ii database. In *CinC* (2004).

14. Douglass, M. J., Cliffford, G. D., Reisner, A. *et al.* De-identification algorithm for free-text nursing notes. *In CinC* (2005).

15. Szarvas, G., Farkas *et al.* State-of-the-art anonymization of medical records using an iterative machine learning framework. *In JAMIA* (2007).

16. Chen, T., Cullen, R. M. & Godwin, M. Hidden markov model using dirichlet process for de-identification. *In JBI* (2015).

17. He, B., Guan, Y., Cheng, J. *et al.* CRFs based de-identification of medical records. *In JBI* (2015).

18. Dehghan, A., Kovacevic *et al.* Combining knowledge-and data-driven methods for de-identification of clinical narratives. *In JBI* (2015).

19. Yang, H. & Garibaldi, J. M. Automatic detection of protected health information from clinic narratives. *J. biomedical informatics* **58**, S30–S38 (2015).

20. Yadav, S., Ekbal *et al.* Deep learning architecture for patient data de-identification in clinical records. In *Proceedings of the clinical natural language processing workshop (ClinicalNLP)*, 32–41 (2016).

21. Pennington, J., Socher, R. & Manning, C. Glove: Global vectors for word representation. In *EMNLP* (2014).

22. Wu, Y., Schuster, M., Chen *et al.* Google's neural machine translation system: Bridging the gap between human and machine translation. *arXiv preprint arXiv:1609.08144* (2016).

23. Glorot, X. & Bengio, Y. Understanding the difficulty of training deep feedforward neural networks. In *AISTATS* (2010).

24. Kingma, D. P. & Ba, J. Adam: A method for stochastic optimization. *arXiv:1412.6980* (2014).

25. Collobert, R., Weston, J., Bottou, L., Karlen, M. & Kavukcuoglu. Natural language processing (almost) from scratch. *In JMLR* (2011).

26. O'malley, K. J. *et al.* Measuring diagnoses: Icd code accuracy. *Heal. services research* **40**, 1620–1639 (2005).
